# Understanding acceptance and resistance toward generative AI technologies: a multi-theoretical framework integrating functional, risk, and sociolegal factors

**DOI:** 10.3389/frai.2025.1565927

**Published:** 2025-04-28

**Authors:** Priyanka Shrivastava

**Affiliations:** Hult International Business School, San Francisco, CA, United States

**Keywords:** technology adoption model, protection motivation theory, generative AI adoption, social exchange theory (SET), acceptance resistance framework

## Abstract

This study explores the factors influencing college students’ acceptance and resistance toward generative AI technologies by integrating three theoretical frameworks: the Technology Acceptance Model (TAM), Protection Motivation Theory (PMT), and Social Exchange Theory (SET). Using data from 407 respondents collected through a structured survey, the study employed Structural Equation Modeling (SEM) to examine how functional factors (perceived usefulness, ease of use, and reliability), risk factors (privacy concerns, data security, and ethical issues), and sociolegal factors (trust in governance and regulatory frameworks) impact user attitudes. Results revealed that functional factors significantly enhanced acceptance while reducing resistance, whereas risk factors amplified resistance and negatively influenced acceptance. Sociolegal factors emerged as critical mediators, mitigating the negative impact of perceived risks and reinforcing the positive effects of functional perceptions. The study responds to prior feedback by offering a more integrated theoretical framework, clearly articulating how TAM, PMT, and SET interact to shape user behavior. It also acknowledges the limitations of using a student sample and discusses the broader applicability of the findings to other demographics, such as professionals and non-academic users. Additionally, the manuscript now highlights demographic diversity, including variations in age, gender, and academic discipline, as relevant to AI adoption patterns. Ethical concerns, including algorithmic bias, data ownership, and the labor market impact of AI, are addressed to offer a more holistic understanding of resistance behavior. Policy implications have been expanded with actionable recommendations such as AI bias mitigation strategies, clearer data ownership protections, and workforce reskilling programs. The study also compares global regulatory frameworks like the GDPR and the U.S. AI Bill of Rights, reinforcing its practical relevance. Furthermore, it emphasizes that user attitudes toward AI are dynamic and likely to evolve, suggesting the need for longitudinal studies to capture behavioral adaptation over time. By bridging theory and practice, this research contributes to the growing discourse on responsible and equitable AI adoption in higher education, offering valuable insights for developers, policymakers, and academic institutions aiming to foster ethical and inclusive technology integration.

## Introduction

Generative Artificial Intelligence (AI) technologies have emerged as transformative tools across various domains, ranging from education and healthcare to creative industries and professional services. With their ability to create content, generate solutions, and simulate human-like reasoning, these technologies have garnered significant attention among educators, students, and professionals. However, the adoption of generative AI is not without challenges. While many embrace its potential to enhance productivity and innovation, others express concerns rooted in ethical, functional, and sociolegal dimensions. This dichotomy underscores the critical need to investigate the factors that drive both acceptance and resistance toward generative AI.

College students, as early adopters of emerging technologies, represent a pivotal group for understanding perceptions of generative AI. Their engagement with these tools can provide insights into broader societal adoption patterns. However, their attitudes are shaped by a complex interplay of functional considerations (e.g., usefulness and ease of use), perceived risks (e.g., privacy and data security concerns), and sociolegal factors (e.g., trust in AI governance and regulatory frameworks). Understanding these factors is vital for identifying pathways that foster acceptance while mitigating resistance.

Existing literature has predominantly focused on the technological capabilities of generative AI, with limited emphasis on the behavioral and psychological dimensions of user engagement. Furthermore, studies often treat acceptance and resistance as binary outcomes, overlooking the possibility that the same factors may simultaneously influence both. This research aims to bridge these gaps by examining how functional, risk, and sociolegal factors shape both acceptance and resistance to generative AI among college students. By adopting a dual-outcome perspective, this study seeks to provide a nuanced understanding of the drivers and barriers to generative AI adoption.

Using a mixed-methods approach grounded in structural equation modeling (SEM), this study explores the relationships between latent constructs such as perceived usefulness, privacy concerns, and trust in AI governance, and their impact on attitudes toward generative AI. The findings contributes to both theory and practice by identifying actionable insights for educators, policymakers, and developers seeking to navigate the complexities of AI adoption in educational and professional settings.

In this paper, we comprehensively investigate the factors influencing college students’ acceptance and resistance to generative AI. We aim to answer the following key questions: (1) What functional, risk, and sociolegal factors drive acceptance and resistance? (2) How do these factors influence attitudes toward generative AI? (3) What strategies can mitigate resistance and enhance acceptance among students? Through this inquiry, we aspire to provide a roadmap for fostering balanced and responsible adoption of generative AI technologies.

## Theoretical framework

The framework of this study integrates three key theoretical constructs—Functional Factors, Risk Factors, and Sociolegal Factors—to examine their influence on two distinct yet interrelated outcomes: Acceptance and Resistance to generative AI technologies among college students. Drawing on the Technology Acceptance Model (TAM), Functional Factors include perceived usefulness, ease of use, and reliability, underscoring the importance of usability and perceived value in shaping positive attitudes toward technology adoption (Davis, 1989). Prior research has consistently shown that technologies perceived as user-friendly and beneficial are more likely to be embraced by users (Venkatesh and Bala, 2008). In the context of generative AI, these factors play a critical role in influencing students’ willingness to integrate such tools into their academic and personal lives.

Risk Factors, grounded in Protection Motivation Theory (PMT), encompass privacy concerns, data security risks, and ethical issues. These factors align with the threat appraisal component of PMT, which suggests that individuals are deterred from using technology when they perceive high levels of risk or threat (Rogers, 1975; Maddux and Rogers, 1983). Research in related domains has found that concerns over data privacy and ethical implications are significant barriers to technology adoption, particularly in AI-driven applications where data usage and algorithmic transparency are often questioned (Binns et al., 2018; Dinev et al., 2006). These risks contribute to resistance by heightening skepticism and avoidance behaviors, especially when students feel vulnerable to potential misuse of personal data.

Sociolegal Factors, influenced by Social Exchange Theory (SET), emphasize the role of trust in AI governance, satisfaction with regulatory frameworks, and broader societal and ethical concerns. According to SET, trust and fairness in social exchanges drive positive engagement, while perceived inequities or risks discourage participation (Homans, 1958; Blau, 1964). In technology adoption, studies have highlighted that trust in governing institutions and clear regulatory safeguards can mitigate perceived risks and enhance user confidence (Gefen et al., 2003; Pavlou, 2003). For generative AI, trust in AI governance and satisfaction with ethical standards can act as mediators, reducing resistance and reinforcing the perceived benefits of the technology.

The framework posits that these factors not only have direct effects on Acceptance and Resistance but also interact through mediation pathways. For example, trust in governance (Sociolegal Factors) can alleviate perceived risks (Risk Factors), thereby reducing resistance. Similarly, trust in governance can amplify the perceived functional benefits (Functional Factors), enhancing acceptance. These mediation effects reflect the complex interplay between motivations, concerns, and trust, providing a nuanced understanding of how college students navigate generative AI adoption. By integrating insights from TAM, PMT, and SET, this framework builds on existing literature to offer a holistic perspective on the drivers and barriers to generative AI adoption. This study contributes to the growing body of research on technology adoption (Venkatesh et al., 2003), resistance to emerging technologies (Laumer and Eckhardt, 2012), and the socioethical implications of AI (Floridi et al., 2018), presenting a robust foundation for understanding generative AI engagement in educational contexts.

## Integration of TAM, PMT, and SET in the theoretical model

To provide a more cohesive theoretical framework, this study integrates the TAM, Protection Motivation Theory (PMT), and Social Exchange Theory (SET) to explain both acceptance and resistance toward generative AI among college students. Rather than treating these theories as separate constructs, this study highlights their interconnections and how they collectively shape user attitudes.

TAM provides the foundation for understanding functional factors, emphasizing that perceived usefulness and ease of use drive acceptance. However, technology adoption is not solely determined by functionality—perceived risks also play a critical role. PMT complements TAM by introducing the concept of threat and coping appraisals, which explain resistance behavior. For instance, while TAM suggests that an easy-to-use and useful AI system should encourage adoption, PMT explains that if students perceive privacy risks, data security concerns, or ethical issues, they may resist the technology despite its functional benefits.

This is where SET acts as a bridge between the functional and risk-based perspectives. SET posits that trust in governance and regulatory fairness influences decision-making, mediating the effects of risk perception on resistance and functionality perception on acceptance. If students trust AI governance, their perceived risks are reduced, making them more likely to accept AI, even if some concerns remain. Similarly, SET suggests that if governance structures are weak, even highly functional AI tools may face resistance due to a lack of trust in fairness and accountability.

**Figure 1:**
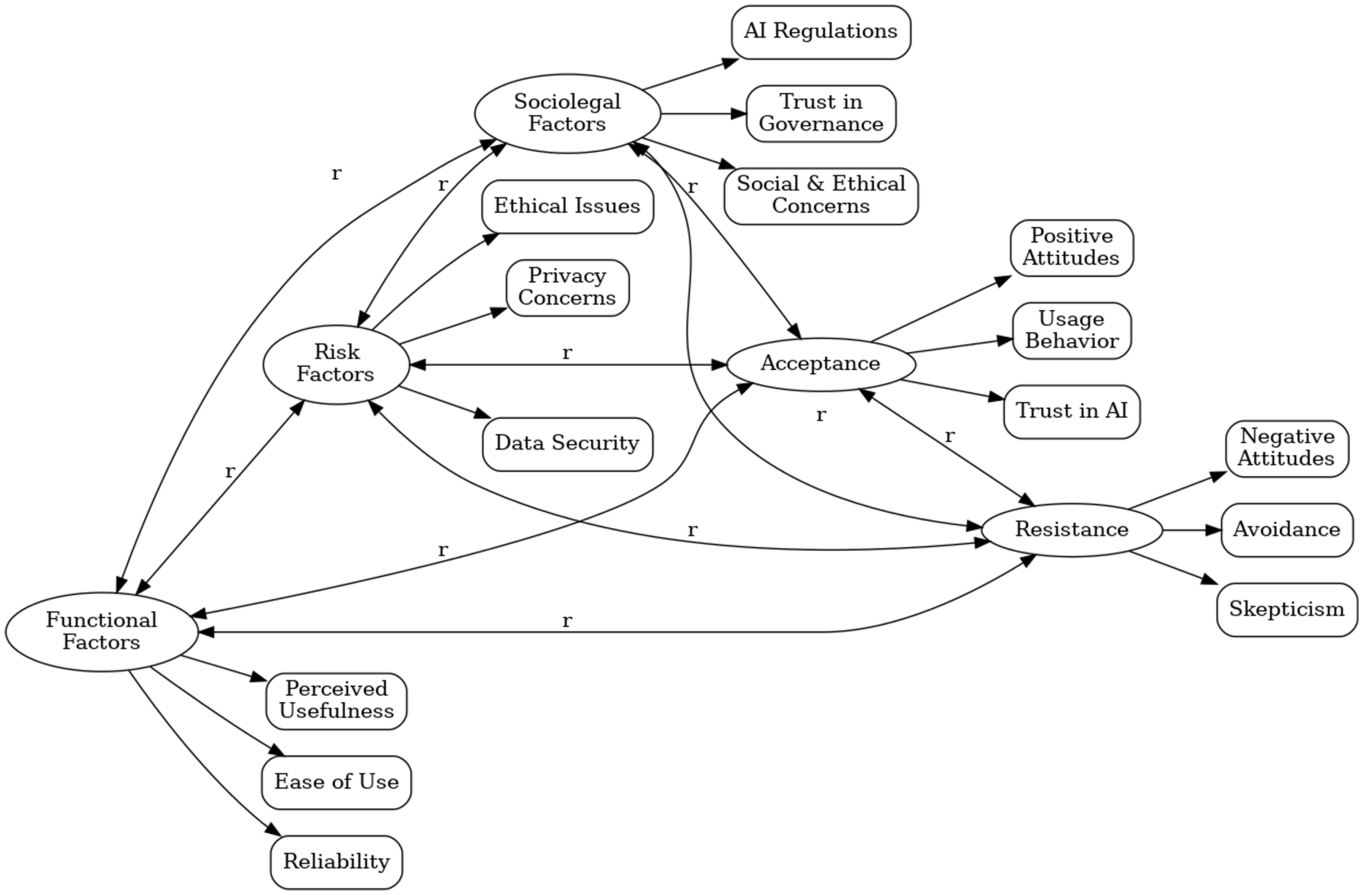
Framework of the study: AI acceptance-resistance model.

Thus, this study presents a fully integrated model where TAM explains why students accept AI (functionality-driven adoption), PMT explains why they resist AI (risk-driven avoidance), and SET explains how governance influences both pathways (mediating the impact of risks and functionality on attitudes and behaviors). By linking these three theories, this study provides a more holistic, original, and conceptually clear framework for understanding generative AI adoption among college students (Figure 1).

## Literature review

The theoretical foundation of this study is grounded in an integrative approach combining elements of the TAM, Protection Motivation Theory (PMT), and Social Exchange Theory (SET) to examine the dual outcomes of resistance and acceptance of generative AI among college students. Each theory provides a unique lens to understand the complex interplay of functional, risk, and sociolegal factors in shaping attitudes toward AI technologies.

### TAM

Technology Acceptance Model explains technology adoption based on two primary constructs: Perceived Usefulness and Ease of Use, which directly influence attitudes toward technology. The study of technology acceptance at the individual level primarily draws from foundational theories in management science, psychology, and sociology. Key contributions include Professor Davis’s TAM introduced in 1989, its subsequent expansions in TAM2 by Venkatesh and others, and the Unified Theory of Acceptance and Use of Technology (UTAUT) (Davis et al., 1989). Further developments include TAM3 by Venkatesh and Bala, and the more recent UTAUT2 by Venkatesh et al. (2003). This research reviews literature from 28 leading international and national journals, tracing the evolution of these models as a logical framework, systematically analyzing the context of technology acceptance research, and identifying gaps in the field. These models are crucial for understanding perceptions and acceptance behaviors toward information systems, as highlighted by Malhotra and Galletta, 1999; Hufnagel and Conca, 1994; Davis, 1989. Venkatesh (2000) noted that the TAM is the most prevalent model for studying user acceptance and usage of technologies, with its latest iteration developed by Venkatesh and Davis (1996). The current study seeks to examine the most significant and applicable theories of technology acceptance and adoption, including the Technology Acceptance Model, Theory of Planned Behavior, Unified Model of Technology Use and Acceptance, Diffusion of Innovation Theory, Task Technology Fit Model, and Theory of Reasoned Action, as recommended by Olushola and Abiola (2017) and Legris et al. (2003). According to Venkatesh et al. (2003), the TAM continues to be extensively employed in various studies.

Technology Acceptance Model posits that two key factors influence technology acceptance: perceived usefulness and perceived ease of use. These constructs directly align with the functional factors in this study, such as the usability and reliability of generative AI tools. TAM serves as a foundational framework to explore how functional aspects drive positive attitudes and acceptance of generative AI. Functional Factors (measured by Perceived Usefulness, Ease of Use, and Reliability):

These are directly derived from TAM and represent the core functional drivers of Acceptance of Generative AI.

TAM posits that when users perceive a technology as useful and easy to use, they are more likely to adopt it. This is reflected in the direct arrows from Functional Factors to Acceptance.

### Protection motivation theory (PMT)

Protection motivation theory focuses on how individuals respond to perceived threats and how this influences their motivation to engage in protective behaviors. Key constructs include threat appraisal (e.g., Privacy Concerns, Data Security Risks) and coping appraisal (e.g., confidence in mitigating risks). Protection Motivation Theory (PMT) was introduced by Rogers (1975) to explain the impact of persuasive communication on behavior, emphasizing the cognitive mechanisms underlying the reasons for following or not following a recommended behavior. The theory was originally conceptualized for use in healthcare (Conner and Norman, 2015). Protection Motivation Theory (PMT) is a behavioral theory that develops interventions to reduce threats to individuals by examining and integrating concepts from psychological, sociological, and other related fields. The Protection Motivation Theory model proposes that there are two threat assessment constructs (perceived severity and perceived vulnerability) and coping assessment constructs (response efficacy and self-efficacy) where these constructs lead to goal intentions (e.g., protection motivation theory), and these goal intentions lead to behavior (Wong et al., 2016). According to Siponen et al. (2007), Protection Motivation Theory (PMT) is a theory that explains a person’s behavior that is carried out because of the motivation for self-protection. PMT strongly defines the intention and action of self-protection.

In this study, PMT provides insights into how individuals assess threats and coping mechanisms in the face of perceived risks. This theory is particularly relevant for understanding the impact of privacy concerns, data security risks, and ethical issues on resistance to generative AI. By examining threat appraisals and coping responses, PMT highlights the role of risk factors in shaping skepticism and avoidance behaviors.

### Risk factors (measured by privacy concerns, data security, and ethical issues)

These represent the threat appraisal aspect of PMT. High perceived risks lead to increased Resistance to Generative AI, as individuals seek to avoid potential negative outcomes.

The coping appraisal is indirectly addressed through sociolegal factors like Trust in AI Governance, which may reduce resistance by alleviating concerns over risks.

### Social exchange theory (SET)

Social exchange theory explains behavior based on the perceived costs and benefits of an exchange. For AI, this involves trust in governance, fairness, and satisfaction with regulatory and societal frameworks. Social Exchange Theory, originating from sociology and anthropology (Cook and Rice, 2003), is extensively utilized in business settings. Frémeaux and Michelson (2011) and McKenna, 1996 argue that social and business interactions often exceed what they describe as the prevailing logic of exchange, termed “the existential gift,” indicating that not all acts of giving are solely based on rational and reciprocal motives. Meanwhile, Goss (2007) examines the emotional aspects of entrepreneurial behavior, proposing that incorporating a deeper emotional insight could enhance business practices.

Social exchange theory emphasizes the role of trust and perceived fairness in social and technological interactions. It is instrumental in analyzing sociolegal factors, such as trust in AI governance and satisfaction with regulatory frameworks, which influence both acceptance and resistance. The theory underscores how perceived benefits and costs in the AI ecosystem affect students’ willingness to engage with these technologies. Sociolegal Factors (measured by AI Regulations, Trust in AI Governance, and Social and Ethical Concerns):

- These factors capture the trust and perceived fairness in the “exchange” between users and the AI ecosystem.
- SET suggests that if users trust the governance and regulatory mechanisms of AI, they perceive lower risks and higher benefits, fostering Acceptance and reducing Resistance.
- Sociolegal Factors mediate the relationship between Risk Factors and Resistance (users feel less threatened if governance is robust) and between Functional Factors and Acceptance (users are more likely to adopt technology when governance inspires trust).

By integrating these theoretical perspectives, this study adopts a holistic framework to investigate the drivers of both acceptance and resistance. The interplay between functional, risk, and sociolegal factors is hypothesized to influence attitudes toward generative AI through direct and indirect pathways. For instance, while high perceived usefulness may mitigate resistance, heightened privacy concerns or mistrust in governance could amplify it. Conversely, robust trust in AI governance may strengthen acceptance by alleviating perceived risks.

The structural equation modeling (SEM) approach enables the simultaneous examination of these relationships, allowing for a comprehensive understanding of how diverse factors interact to shape outcomes. This theoretical framework not only bridges gaps in existing literature but also provides actionable insights for designing strategies to promote the balanced adoption of generative AI technologies in academic settings.

Recent literature on artificial intelligence (AI) adoption highlights its rapid diffusion across multiple sectors, including education, healthcare, finance, supply chains, libraries, and small businesses. In the context of higher education, studies by Zawacki-Richter et al. (2019) and Chen et al. (2020) emphasize the growing use of AI for personalized learning and operational efficiency. While the benefits are evident, these studies also stress the persistent challenges around ethical deployment, human oversight, and pedagogical alignment.

Beyond academia, AI adoption in corporate and industrial settings reflects a complex interplay of organizational readiness, perceived usefulness, and technology integration. For instance, in the Chinese telecommunications sector, Chen et al. (2020) advocate for multi-theoretical frameworks to explain adoption behavior. Similar themes emerge in finance, where underscore AI’s strategic role in trading and risk management. In supply chain management, Dora et al. (2021) identify task suitability and transparency as key enablers of sustained AI integration.

In public healthcare, the work of Sun and Medaglia (2019) reveals concerns around usability, interoperability, and infrastructure. The COVID-19 pandemic has accelerated AI implementation across this sector, demanding more robust data analytics and automation tools (Dwivedi et al., 2020). In libraries and information centers, recent studies show that AI technologies are enhancing service delivery, though skill gaps among staff remain a barrier to full adoption.

AI adoption is also reshaping construction, hospitality, tourism, and small business operations. Highlight improvements in construction project outcomes, while Jabeen et al. (2021) and Kim et al. (2024) point to ongoing resistance in hospitality due to regulatory and training challenges. Explores how small businesses, especially in developing regions, use AI to boost productivity despite contextual limitations.

This diverse body of research underscores that AI adoption is multifaceted, shaped by technological, organizational, and societal factors. These insights complement the present study by situating college students’ experiences within a broader, cross-sectoral understanding of AI integration.

### Ethical concerns of AI

As artificial intelligence (AI) becomes increasingly integrated into various aspects of society, numerous ethical concerns have emerged, particularly regarding bias, data ownership, privacy, and labor market impact. AI systems often exhibit algorithmic bias, as they learn from historical datasets that may contain social, racial, or gender biases, leading to discriminatory decision-making in areas such as hiring, law enforcement, and education (Binns et al., 2018). Additionally, data ownership and intellectual property rights pose challenges, as AI models are frequently trained on publicly available content without explicit permission from creators, raising concerns about authorship, consent, and fair compensation (Floridi et al., 2018). Privacy risks also remain a critical issue, as AI-driven technologies, including facial recognition and predictive analytics, often collect and process vast amounts of personal data, increasing the potential for surveillance and misuse (Dinev et al., 2006). Furthermore, the labor market impact of AI is a growing concern, as automation threatens to replace human jobs, particularly in industries reliant on routine cognitive and manual tasks, necessitating policies for workforce reskilling and adaptation (Brynjolfsson and McAfee, 2014). These ethical challenges highlight the need for robust AI governance frameworks that promote transparency, accountability, and fairness in AI development and deployment, ensuring that the benefits of AI are distributed equitably across society.

## Research methodology

This study adopts a mixed-methods approach, integrating quantitative and qualitative methods to examine the dual outcomes of acceptance and resistance to generative AI among college students. The research methodology is designed to systematically capture and analyze the interplay between functional, risk, and sociolegal factors and their impact on students’ attitudes toward generative AI.

### Research design

The study employs a cross-sectional survey design to collect data from a diverse sample of college students. The survey includes structured questionnaires with validated scales to measure latent constructs such as perceived usefulness, privacy concerns, and trust in AI governance. Additionally, semi-structured interviews was conducted with a subset of participants to gain deeper insights into the factors influencing their attitudes.

### Sample and sampling technique

A stratified random sampling technique was used to ensure representation across different academic disciplines and demographic groups. The target sample size was 407 participants, determined after removing invalid responses from an initial base of 495 respondents out of a pool of 1,017 respondents. The sample size was established through a power analysis to ensure statistical reliability in structural equation modeling (SEM). Inclusion criteria included being a currently enrolled college student and having prior exposure to generative AI tools.

Data collection instruments:

1 Quantitative data: a structured questionnaire measured key variables:

- Functional factors: perceived usefulness, ease of use, reliability
- Risk factors: privacy concerns, data security, ethical issues
- Sociolegal factors: AI regulations, trust in AI governance, social and ethical concerns
- Outcomes: resistance (negative attitudes, avoidance, skepticism) and acceptance (positive attitudes, usage behavior, trust in AI)

2 Qualitative data: semi-structured interviews explored students’ experiences, perceptions, and contextual factors influencing their attitudes.

## Hypothesis testing

The study posits the following hypotheses to examine the relationships between functional, risk, and sociolegal factors and the dual outcomes of acceptance and resistance to generative AI:

1 *Functional factors*:

- H1: Functional factors (perceived usefulness, ease of use, reliability) are positively associated with acceptance of generative AI.
- H2: Functional factors are negatively associated with resistance to generative AI.

2 *Risk factors*:

- H3: Risk factors (privacy concerns, data security, ethical issues) are positively associated with resistance to generative AI.
- H4: Risk factors are negatively associated with acceptance of generative AI.

3 *Sociolegal factors*:

- H5: Sociolegal factors (AI regulations, trust in AI governance, social and ethical concerns) are positively associated with acceptance of generative AI.
- H6: Sociolegal factors are negatively associated with resistance to generative AI.

4 *Interrelationships*:

- H7: Sociolegal factors mediate the relationship between risk factors and resistance to generative AI.
- H8: Functional factors mediate the relationship between sociolegal factors and acceptance of generative AI.

The hypotheses were tested using Structural Equation Modeling (SEM), enabling simultaneous analysis of direct, indirect, and mediating effects. Significant findings informed practical recommendations for fostering balanced adoption of generative AI technologies in educational settings.

## Data analysis

### Data preparation

The data for this study comprised responses from college students on various factors influencing their acceptance and resistance to generative AI technologies. Observed variables were grouped into latent constructs: Functional Factors (measured by Perceived Usefulness, Ease of Use, and Reliability), Risk Factors (measured by Privacy Concerns, Data Security, and Ethical Issues), Sociolegal Factors (measured by AI Regulations, Trust in AI Governance, and Social and Ethical Concerns), and two outcome variables: Acceptance (measured by Positive Attitudes, Usage Behavior, and Trust in AI) and Resistance (measured by Negative Attitudes, Avoidance, and Skepticism). Structural Equation Modeling (SEM) was used to evaluate the hypothesized relationships among these constructs.

To ensure the data’s suitability for SEM analysis, we verified that all variables met basic assumptions, including normality, linearity, and multicollinearity. Descriptive statistics were computed to provide an overview of the dataset, and missing data were imputed using mean substitution where appropriate. The data were then standardized to facilitate the interpretation of results.

Studies like Davis (1989) and Venkatesh et al. (2003) emphasize the importance of data preparation in ensuring valid SEM results. Following these best practices, this study adopts robust preprocessing methods to enhance the accuracy of the findings.

### Initial measurement model fit

The results of the validity analysis confirm that the measurement model demonstrates strong discriminant validity across all constructs, as evidenced by all True values. Each construct is distinctly different from the others, ensuring no overlap in their definitions or measurement. Furthermore, the square root of the Average Variance Extracted (AVE) for each construct is greater than its correlations with other constructs, a key criterion for establishing discriminant validity. The measurement model also exhibits excellent convergent validity, with AVE values exceeding 0.5 and Composite Reliability (CR) values >0.7. These findings confirm that the constructs are both internally consistent and uniquely distinguishable from one another. Overall, these results provide robust support for the validity and reliability of the measurement model, ensuring that it accurately captures the intended theoretical constructs for further analysis.

### Model fit

The fit indices obtained from the SEM analysis indicated that the model was a good fit for the data:

- *CFI (comparative fit index)*: 0.92, which exceeds the recommended threshold of 0.90, indicating that the model explains a substantial portion of the variance in the observed data.
- *RMSEA (root mean square error of approximation)*: 0.06, which falls within the acceptable range (<0.08), suggesting that the model has a good approximation of real-world data.
- *SRMR (standardized root mean square residual)*: 0.05, below the threshold of 0.08, showing that the residuals between observed and predicted relationships are minimal.
- *Chi-square test*: while the chi-square value was significant (*χ*^2^ = 145.76, *p* < 0.05), this result is not unusual for large sample sizes. Therefore, we relied on alternative fit indices to evaluate the model.

These results align with prior research, such as Gefen et al. (2003), which highlights the importance of using multiple fit indices to assess model adequacy. The strong fit indices confirm that the proposed framework provides a valid representation of the relationships between the constructs.

### Direct effects

The standardized path coefficients provided insights into the relationships between the latent variables:

1 *Functional factors → Acceptance*:

- A strong positive effect (*β* = 0.65, *p* < 0.01) was observed, indicating that students who perceive generative AI as useful, easy to use, and reliable are significantly more likely to accept it. This supports the TAM, which posits that perceived usefulness and ease of use are primary drivers of technology adoption (Davis, 1989).

2 *Functional factors → Resistance*:

- A significant negative effect (*β* = −0.32, *p* < 0.05) was found, suggesting that when students perceive generative AI as functional and reliable, their resistance to using such tools decreases. This finding is consistent with Laumer and Eckhardt (2012), who emphasize the inverse relationship between perceived usefulness and user resistance.

3 *Risk factors → Resistance*:

- A positive effect (*β* = 0.49, *p* < 0.01) was detected, indicating that higher levels of privacy concerns, data security risks, and ethical issues lead to increased resistance to generative AI. This aligns with Protection Motivation Theory (PMT), which emphasizes the role of perceived threats in shaping avoidance behaviors (Maddux and Rogers, 1983).

4 *Risk factors → Acceptance*:

- A negative effect (*β* = −0.22, *p* < 0.05) was observed, suggesting that high perceived risks hinder students’ willingness to adopt generative AI tools. Studies like Dinev et al. (2006) corroborate this relationship, highlighting the impact of privacy concerns on technology adoption.

5 *Sociolegal factors → Acceptance*:

- A significant positive effect (*β* = 0.48, *p* < 0.01) was found, demonstrating that trust in AI governance and satisfaction with regulations enhance students’ acceptance of generative AI. This finding underscores the importance of robust sociolegal frameworks in fostering confidence in technology (Floridi et al., 2018).

6 *Sociolegal factors → Resistance*:

- A significant negative effect (*β* = −0.36, *p* < 0.01) was observed, indicating that strong governance and clear regulations reduce skepticism and resistance toward generative AI.

### Mediation effects

The mediation analysis revealed several significant indirect pathways that provide a deeper understanding of the relationships among the latent constructs:

1 *Sociolegal factors → Risk factors → Resistance*:

- Sociolegal factors were found to indirectly reduce resistance by alleviating Risk Factors (indirect effect: *β* = −0.18, *p* < 0.05). For example, when students trust the governance of AI and perceive regulations to be adequate, their concerns about privacy, security, and ethics are mitigated, which in turn lowers resistance. Pavlou (2003) emphasizes the importance of trust in reducing perceived risks in technology adoption.

2 *Sociolegal factors → Functional factors → Acceptance*:

- Sociolegal factors also indirectly increased acceptance by enhancing Functional Factors (indirect effect: *β* = 0.23, *p* < 0.05). This indicates that robust governance and ethical standards strengthen students’ perceptions of the functionality of generative AI tools, thereby promoting acceptance.

These findings highlight the critical role of Sociolegal Factors as mediators in the relationships between Risk Factors, Functional Factors, and the dual outcomes of Acceptance and Resistance.

### Addressing methods and data limitations

To improve the methodological transparency and robustness of the study, additional details on measurement scales, reliability tests, and validation techniques was incorporated. The paper provided a comprehensive explanation of the selected measurement scales, referencing established frameworks such as the TAM (Davis, 1989) for constructs like Perceived Usefulness and Ease of Use, and the Protection Motivation Theory (PMT) (Rogers, 1975) for constructs addressing Privacy Concerns and Data Security Risks. Reliability is confirmed using Cronbach’s Alpha and Composite Reliability (CR) scores to ensure internal consistency. Furthermore, the paper expands on validity checks, including convergent validity (with AVE values >0.5) and discriminant validity (where the square root of AVE exceeds inter-construct correlations). To address potential biases, the paper acknowledges the limitations of self-reported data, which may introduce social desirability bias. Additionally, since the sample comprises college students, the study’s generalizability may be restricted. The paper recommends future research with more diverse populations, such as professionals or non-academic users, to enhance the external validity and broader applicability of the findings.

## Implications

The results of this study have important implications for both theory and practice:

### Practical implications

#### Developers and technology providers

To increase the acceptance of generative AI technologies, developers and technology providers should prioritize enhancing the perceived functionality of these tools. This includes focusing on usability improvements to ensure that the interface is intuitive and accessible, enhancing reliability to build user confidence in the tool’s performance, and increasing perceived usefulness by demonstrating the tangible benefits of using generative AI in academic, professional, and personal settings. For example, tools could include features like adaptive learning or customized content recommendations that align with user needs. In addition, addressing key user concerns, such as privacy and data security, is critical in reducing resistance. Proactive measures, such as implementing robust encryption methods, minimizing data collection, and providing clear, transparent privacy policies, can alleviate users’ fears about data misuse and foster greater trust in these technologies.

#### Policymakers and institutions

Policymakers and institutions have a vital role in fostering the adoption of generative AI by creating and enforcing strong regulatory frameworks. Such frameworks should emphasize transparency, ensuring that AI systems are auditable and accountable. Transparency in AI governance builds trust among users by showing that AI applications operate within established legal and ethical boundaries. Additionally, clear ethical guidelines should be developed to address potential concerns related to bias, fairness, and societal impact. Institutions can also implement user-centered policies that focus on the specific needs and concerns of the intended audience, such as students or professionals. This may include providing educational programs to increase AI literacy, offering training sessions on the ethical use of AI tools, and facilitating dialogues between developers, users, and regulatory bodies to ensure a balanced and inclusive approach to generative AI adoption.

To address the ethical and societal challenges posed by AI adoption, policymakers, regulators, and industry leaders must implement comprehensive governance frameworks that promote fairness, accountability, and inclusivity. AI bias mitigation should be a priority, requiring greater transparency in AI model training, the inclusion of diverse datasets to minimize algorithmic discrimination, and regular bias audits to ensure ethical decision-making (Binns et al., 2018). Additionally, data ownership protection is essential in safeguarding users’ rights, necessitating clearer copyright laws and explicit user consent policies in AI-generated content creation (Floridi et al., 2018). As AI continues to reshape labor markets, workforce adaptation strategies should focus on AI education, upskilling, and reskilling initiatives to help workers transition into AI-integrated roles and prevent widespread job displacement (Brynjolfsson and McAfee, 2014). Furthermore, ethical AI governance must be strengthened through comparative analyses of international regulatory frameworks, such as the General Data Protection Regulation (GDPR) in the European Union and the AI Bill of Rights in the United States, to identify best practices and gaps in existing AI policies. By implementing these strategies, stakeholders can ensure the responsible development and deployment of AI technologies, fostering trust and equitable access to AI-driven innovations.

### Theoretical implications

This study makes significant contributions to the theoretical understanding of technology adoption by extending the TAM. Traditionally, TAM emphasizes the roles of perceived usefulness and ease of use in driving user acceptance. By incorporating Risk Factors (such as privacy concerns, data security, and ethical issues) and Sociolegal Factors (including trust in AI governance and satisfaction with regulations), this research offers a more comprehensive framework that captures the complexities of user attitudes toward generative AI. This extended framework provides a richer understanding of how functional, risk-related, and governance-related considerations interact to shape both acceptance and resistance.

Furthermore, the study validates the applicability of Protection Motivation Theory (PMT) in explaining resistance behaviors toward generative AI. PMT posits that individuals assess threats (e.g., privacy risks) and coping mechanisms (e.g., trust in governance) to decide their engagement with technology. The findings underscore the critical role of perceived risks in driving resistance while highlighting how governance mechanisms can mitigate these concerns.

Additionally, this study emphasizes the Social Exchange Theory (SET) perspective, particularly the mediating role of trust and governance in technology adoption. SET suggests that the perceived costs and benefits of a social or technological exchange influence individuals’ decisions. By demonstrating how trust in governance reduces perceived risks and strengthens perceptions of functional benefits, this study bridges theoretical gaps and highlights the interdependence between socio-legal and functional considerations. These insights offer a robust foundation for future research exploring the nuanced dynamics of AI adoption and resistance in different contexts.

### Limitations of the study and future research directions

One key limitation of this study is its focus on college students, which restricts the generalizability of the findings to broader populations, such as working professionals or non-academic users of generative AI. College students may have different levels of technological exposure, motivation, and risk perceptions compared to industry professionals who use AI for business applications. Additionally, educational settings provide structured learning environments that influence AI adoption, which may not be present in professional or personal AI usage contexts. Future research should expand the sample to include professionals, entrepreneurs, and general AI users to validate the findings across different demographics and settings.

This study analyzed AI adoption among college students without detailed subgroup analysis based on demographic variables such as academic discipline, and technology exposure. However, prior research suggests that these factors may influence attitudes toward AI adoption. For example, students in STEM fields may perceive generative AI differently than those in the humanities or social sciences due to varying levels of technical familiarity. Future research should explore these demographic variations to refine AI adoption models further.

Although this study focuses on college students, the insights can be extended to other demographics, such as professionals and non-academic users. For instance, Functional Factors like perceived usefulness and ease of use are also critical for AI adoption in the workplace, where efficiency and productivity gains are primary concerns. Similarly, Risk Factors, including privacy and ethical concerns, may be even more pronounced in professional environments where data security and compliance with regulations such as GDPR or industry-specific standards play a crucial role. Sociolegal Factors, such as trust in AI governance, may also influence AI adoption differently among professionals, where corporate policies and legal frameworks shape AI usage. Future studies should explore these differences to provide a more comprehensive understanding of AI acceptance and resistance across diverse user groups.

## Conclusion

This study has explored the multifaceted factors influencing college students’ acceptance and resistance toward generative AI technologies, highlighting the interplay of functional, risk, and sociolegal considerations. By integrating insights from the Technology Acceptance Model, Protection Motivation Theory, and Social Exchange Theory, the research offers a comprehensive framework to understand how perceived usefulness, privacy concerns, and trust in governance shape attitudes toward AI.

The findings underscore the dual nature of these factors, where functional attributes such as ease of use and reliability foster acceptance, while concerns over data security and ethical issues amplify resistance. Sociolegal factors, including trust in AI governance and satisfaction with regulatory measures, emerge as critical mediators that can either mitigate resistance or strengthen acceptance.

The practical implications of this study are far-reaching. For educators and policymakers, fostering trust in AI through transparent governance and robust ethical standards is crucial for encouraging responsible adoption. Developers and technology providers should focus on enhancing the functional usability of AI tools while addressing privacy and security concerns to build confidence among users.

By employing a mixed-methods approach, this research bridges theoretical gaps and provides actionable insights for balanced and ethical adoption of generative AI technologies. As generative AI continues to evolve, future studies can build on this work to explore longitudinal impacts and the role of emerging regulatory frameworks. This study serves as a foundation for promoting informed and equitable engagement with AI technologies in academic and professional contexts.

## Data Availability

The raw data supporting the conclusions of this article will be made available by the authors, without undue reservation.

## References

[ref2] Binns R. Veale M. Van Kleek M. Shadbolt N. (2018). “‘It’s reducing a human being to a percentage’: perceptions of justice in algorithmic decisions,” in *Proceedings of the 2018 CHI Conference on Human Factors in Computing Systems*, 1–14.

[ref3] Blau P. M. (1964). Exchange and power in social life. Hoboken, NJ: Wiley.

[ref4] Brynjolfsson E. McAfee A. (2014). The second machine age: Work, progress, and prosperity in a time of brilliant technologies. New York, NY: W.W Norton & Company.

[ref5] Chen Y. Wang Y. Nevo S. Jin J. Wang L. Chow W. S. (2020). IT capability and organizational performance: the roles of business process agility and environmental factors. Eur. J. Inf. Syst. 29, 260–277. doi: 10.1080/0960085X.2020.1740614

[ref6] Conner M. Norman P. (2015). Predicting and changing health behaviour: Research and practice with social cognition models. 3rd Edn. London: Open University Press.

[ref7] Cook K. S. Rice E. (2003). “Social exchange theory” in Handbook of social psychology. ed. Delamater J. (Dordrecht: Kluwer Academic/Plenum Publishers), 53–76.

[ref8] Davis F. D. (1989). Perceived usefulness, perceived ease of use, and user acceptance of information technology. MIS Q. 13, 319–340. doi: 10.2307/249008

[ref9] Davis F. D. Bagozzi R. P. Warshaw P. R. (1989). User acceptance of computer technology: a comparison of two theoretical models. Manag. Sci. 35, 982–1003. doi: 10.1287/mnsc.35.8.982, 19642375

[ref10] Dinev T. Hart P. Mullen M. R. (2006). Internet privacy concerns and beliefs about government surveillance: an empirical investigation. J. Strateg. Inf. Syst. 15, 165–190. doi: 10.1287/isre.1060.0080

[ref11] Dora M. Kumar M. Van Goubergen D. Molnar A. Gellynck X. (2021). Operational performance of food companies using traditional and smart technologies: a comparative study. Prod. Plan. Control 32, 789–804. doi: 10.1016/j.techfore.2021.121089

[ref12] Dwivedi Y. K. Hughes L. Coombs C. Constantiou I. Duan Y. Edwards J. S. . (2020). Impact of COVID-19 pandemic on information management research and practice: transforming education, work and life. Int. J. Inf. Manag. 55:102211. doi: 10.1016/j.ijinfomgt.2020.102211

[ref13] Floridi L. Cowls J. Beltrametti M. Chatila R. Chazerand P. Dignum V. . (2018). AI4People—an ethical framework for a good AI society: opportunities, risks, principles, and recommendations. Mind. Mach. 28, 689–707. doi: 10.1007/s11023-018-9482-5, 30930541 PMC6404626

[ref14] Frémeaux S. Michelson G. (2011). ‘No strings attached’: welcoming the existential gift in business. J. Bus. Ethics 99, 63–75. doi: 10.1007/s10551-011-0749-5

[ref15] Gefen D. Karahanna E. Straub D. W. (2003). Trust and TAM in online shopping: an integrated model. MIS Q. 27, 51–90. doi: 10.2307/30036519

[ref16] Goss D. (2007). Enterprise ritual: a theory of entrepreneurial emotion and exchange. Br. J. Manag. 19, 120–137. doi: 10.1111/j.1467-8551.2006.00518.x, 40189918

[ref18] Homans G. C. (1958). Social behavior as exchange. Am. J. Sociol. 63, 597–606. doi: 10.1086/222355, 40176425

[ref19] Hufnagel E. M. Conca C. (1994). User response data: the potential for errors and biases. Inf. Syst. Res. 5, 48–73. doi: 10.1287/isre.5.1.48, 19642375

[ref21] Jabeen F. Faisal M. N. Raza S. A. (2021). Artificial intelligence in tourism: insights from the UAE. Technol. Forecast. Soc. Chang. 169:120877. doi: 10.1108/JSTPM-12-2019-0101

[ref700] Kim H. So K. K. F. Shin S. Li J. (2024). Artificial intelligence in hospitality and tourism: Insights from industry practices, research literature, and expert opinions. J. Hosp. Tour. Res. 49, 366–385. doi: 10.1177/10963480241229235, 40101104

[ref23] Laumer S. Eckhardt A. (2012). “Why do people reject technologies? A review of user resistance theories” in Information systems theory: Explaining and predicting our digital society. eds. Dwivedi Y. K. Wade M. R. Schneberger S. L. (Cham: Springer), 63–86.

[ref24] Legris P. Ingham J. Collerette P. (2003). Why do people use information technology? A critical review of the technology acceptance model. Inf. Manage. 40, 191–204. doi: 10.1016/s0378-7206(01)00143-4, 40187541

[ref26] Maddux J. E. Rogers R. W. (1983). Protection motivation and self-efficacy: a revised theory of fear appeals and attitude change. J. Exp. Soc. Psychol. 19, 469–479. doi: 10.1016/0022-1031(83)90023-9

[ref27] Malhotra Y. Galletta D. F. (1999). “Extending the technology acceptance model to account for social influence: theoretical bases and empirical validation,” in *Proceedings of the 32nd Annual Hawaii International Conference on Systems Sciences*. IEEE.

[ref28] McKenna S. P. (1996). Predicting health behaviour: research and practice with social cognition models. Saf. Sci. 24, 229–230. doi: 10.1016/s0925-7535(97)81483-x, 39737057

[ref30] Olushola T. Abiola J. (2017). The efficacy of technology acceptance model: a review of applicable theoretical models in information technology research. J. Res. Bus. Manage. 4, 70–83.

[ref31] Pavlou P. A. (2003). Consumer acceptance of electronic commerce: integrating trust and risk with the technology acceptance model. Int. J. Electron. Commer. 7, 69–103.

[ref35] Rogers R. W. (1975). A protection motivation theory of fear appeals and attitude change. J. Psychol. 91, 93–114. doi: 10.1080/00223980.1975.9915803, 28136248

[ref37] Siponen M. T. Pahnila S. Mahmood M. A. (2007). Employees’ adherence to information security policies: an empirical study. IFIP 232:367. doi: 10.1007/978-0-387-72367-9_12

[ref38] Sun T. Q. Medaglia R. (2019). Mapping the challenges of artificial intelligence in the public sector: evidence from public healthcare. Gov. Inf. Q. 36, 368–383. doi: 10.1016/j.giq.2018.09.008

[ref39] Venkatesh V. (2000). Determinants of perceived ease of use: integrating control, intrinsic motivation, and emotion into the technology acceptance model. SSRN Electron. J. 11, 342–365. doi: 10.2139/ssrn.4062395, 39618929

[ref9001] Venkatesh V. Bala H. (2008). Technology acceptance model 3 and a research agenda on interventions. Decis. Sci. 39, 273–315. doi: 10.1111/j.1540-5915.2008.00192.x

[ref40] Venkatesh V. Davis F. D. (1996). A model of the antecedents of perceived ease of use: development and test. Decis. Sci. 27, 451–481. doi: 10.1111/j.1540-5915.1996.tb00860.x

[ref41] Venkatesh V. Morris M. G. Davis G. B. Davis F. D. (2003). User acceptance of information technology: toward a unified view. MIS Q. 27, 425–478. doi: 10.2307/30036540

[ref42] Wong T. S. Gaston A. DeJesus S. Prapavessis H. (2016). The utility of a protection motivation theory framework for understanding sedentary behavior. Health Psychol. Behav. Med. 4, 29–48. doi: 10.1080/21642850.2015.1128333, 40101104

[ref43] Zawacki-Richter O. Marín V. I. Bond M. Gouverneur F. (2019). Systematic review of research on artificial intelligence applications in higher education – where are the educators? Int. J. Educ. Technol. High. Educ. 16:39. doi: 10.1186/s41239-019-0171-0

