# Correction: Understanding acceptance and resistance toward generative AI technologies: a multi-theoretical framework integrating functional, risk, and sociolegal factors

**DOI:** 10.3389/frai.2025.1751706

**Published:** 2026-02-12

**Authors:** Priyanka Shrivastava

**Affiliations:** Hult International Business School, San Francisco, CA, United States

**Keywords:** technology adoption model, protection motivation theory, generative AI adoption, social exchange theory (SET), acceptance resistance framework

The references for: Aydin and Sirkeci (2024), Hajj and Hammoud (2023), Ikpe, (2023), Jha, S (2023), Li (2024), Modgil, et al. (2021), Rahmani (2023), Rane et al. (2024) Regona et al. (2022) and Schepart et al. (2023), were erroneously written as:

Aydin, G., and Sirkeci, I. (2024). Artificial intelligence adoption in hospitality and tourism: opportunities and constraints. *J. Tour. Hosp. Manage*. 12, 33–47.

Hajj, S., and Hammoud, R. (2023). Artificial intelligence in finance: strategic adoption and risk implications. *J. Financ. Innov*. 9, 118–134.

Ikpe, E. (2024). Artificial intelligence in small businesses: opportunities and adoption barriers in emerging economies. *Int. J. Small Bus. Technol*. 10, 56–72.

Jha, S. (2023). Leveraging artificial intelligence in library systems: a review of current practices and future directions. *J. Librar. Inf. Technol*. 43, 110–120.

Li, X. (2024). AI-powered transformation in academic libraries: case studies and future prospects. *Library Manage. Rev*. 45, 25–39.

Modgil, S., Gupta, S., and Nandy, A. (2021). AI adoption in supply chain management: an empirical examination of enablers. *J. Enterp. Inf. Manag*. 34, 1450–1471.

Rahmani, M. (2023). Digital innovation in libraries: exploring artificial intelligence as a driver of service transformation. *Int. J. Inf. Sci*. 19, 201–215.

Rane, A., Patel, D., and Shukla, M. (2024). Artificial intelligence in construction safety and risk management. *Constr. Technol. Today* 18, 78–90.

Regona, D., Tan, R., and Lucero, J. (2022). AI-enhanced project management in the construction industry: a Philippine case study. *Asian J. Eng. Technol. Innov*. 10, 145–162.

Schepart, H., Chen, C. A., and Davis, J. (2023). AI in public health systems: infrastructure, usability, and implementation challenges. *J. Health Inf*. 28, 9–23.

They will be removed from the article.

Aydin and Sirkeci (2024) should be replaced with: Kim, H., So, K. K. F., Shin, S., and Li, J. (2024). Artificial intelligence in hospitality and tourism: Insights from industry practices, research literature, and expert opinions. *J. Hosp. Tour. Res*. 49, 366–385. doi: 10.1177/10963480241229235.

The original version of this article has been updated.

