# Supplementary material for: Understanding acceptance and resistance toward generative AI technologies: a multi-theoretical framework integrating functional, risk, and sociolegal factors
Source: Front Artif Intell. 2025 Apr 28;8:1565927. doi: 10.3389/frai.2025.1565927 (PMC12066764; doi:10.3389/frai.2025.1565927)
Supplement: Supplementary file 1 [file Data_Sheet_1.docx]

Appendix 1

Structured Questionnaire:

**Academic Study on GEN AI tools**

1.Responses of Gen AI tools help me write my assignment.

- Strongly Disagree
- Disagree
- Neutral
- Agree
- Strongly Agree

2.Responses of Gen AI help me to score better.

- Strongly Disagree
- Disagree
- Neutral
- Agree
- Strongly Agree

3.Responses of Gen AI such as ChatGPT do not aid me in increasing my knowledge.

- Strongly Disagree
- Disagree
- Neutral
- Agree
- Strongly Agree

4.I believe responses by Gen AI improve my academic performance.

- Strongly Disagree
- Disagree
- Neutral
- Agree
- Strongly Agree

5.I am confident in putting responses by Gen AI in my assignments/ academic deliverables.

- Strongly Disagree
- Disagree
- Neutral
- Agree
- Strongly Agree

6. I find it convenient that I can use Gen AI wherever I want.

- Strongly Disagree
- Disagree
- Neutral
- Agree
- Strongly Agree

7. I can get my responses from Gen AI without having to wait for someone else to be available.

- Strongly Disagree
- Disagree
- Neutral
- Agree
- Strongly Agree

8.I believe human bias does not affect the responses by Gen AI.

- Strongly Disagree
- Disagree
- Neutral
- Agree
- Strongly Agree

9.Responses by Gen AI do not support any vested interest (political/social/economical).

- Strongly Disagree
- Disagree
- Neutral
- Agree
- Strongly Agree

10. Responses by Gen AI are opinionated.

- Strongly Disagree
- Disagree
- Neutral
- Agree
- Strongly Agree

11.I can easily understand responses produced by Gen AI.

- Strongly Disagree
- Disagree
- Neutral
- Agree
- Strongly Agree

12.The responses of Gen AI are vague and complex.

- Strongly Disagree
- Disagree
- Neutral
- Agree
- Strongly Agree

13.My knowledge on specific topics has increased after using Gen AI.

- Strongly Disagree
- Disagree
- Neutral
- Agree
- Strongly Agree

14.Responses of Gen AI are generic and are not backed by research & data.

- Strongly Disagree
- Disagree
- Neutral
- Agree
- Strongly Agree

15.The responses of Gen AI meet my standards.

- Strongly Disagree
- Disagree
- Neutral
- Agree
- Strongly Agree

16.Gen AI tools generate responses to my benefit.

- Strongly Disagree
- Disagree
- Neutral
- Agree
- Strongly Agree

17.Gen AI tools do not jeopardize my privacy.

- Strongly Disagree
- Disagree
- Neutral
- Agree
- Strongly Agree

18.Gen AI tools do not share my data with third parties.

- Strongly Disagree
- Disagree
- Neutral
- Agree
- Strongly Agree

19.Responses of Gen AI tools are free from human errors.

- Strongly Disagree
- Disagree
- Neutral
- Agree
- Strongly Agree

20.Human errors cannot manipulate the contents of Gen AI tools.

- Strongly Disagree
- Disagree
- Neutral
- Agree
- Strongly Agree

21.Gen AI tools are incapable to answer all my questions.

- Strongly Disagree
- Disagree
- Neutral
- Agree
- Strongly Agree

22.I can find answers to all my questions in Gen AI tools.

- Strongly Disagree
- Disagree
- Neutral
- Agree
- Strongly Agree

23.I believe humans can answer questions better than Gen AI.

- Strongly Disagree
- Disagree
- Neutral
- Agree
- Strongly Agree

24.I believe Gen AI tools are faster in answering my questions compared to other resources/platforms.

- Strongly Disagree
- Disagree
- Neutral
- Agree
- Strongly Agree

25.I believe Gen AI tools are complex AI beyond human comprehension.

- Strongly Disagree
- Disagree
- Neutral
- Agree
- Strongly Agree

26.Gen AI tools are vague and difficult systems to understand.

- Strongly Disagree
- Disagree
- Neutral
- Agree
- Strongly Agree

27.Responses of Gen AI tools are similar for everyone irrespective of differences in their personal preferences and characteristics.

- Strongly Disagree
- Disagree
- Neutral
- Agree
- Strongly Agree

28.The responses of Gen AI tools do not consider my personal interests.

- Strongly Disagree
- Disagree
- Neutral
- Agree
- Strongly Agree

29.Responses of Gen AI tools are not customized based on individuals’ backgrounds.

- Strongly Disagree
- Disagree
- Neutral
- Agree
- Strongly Agree

30.Responses of Gen AI tools are accurate and precise.

- Strongly Disagree
- Disagree
- Neutral
- Agree
- Strongly Agree

31.Gen AI tools produce false and misleading information.

- Strongly Disagree
- Disagree
- Neutral
- Agree
- Strongly Agree

32.Responses by Gen AI tools lack novelty.

- Strongly Disagree
- Disagree
- Neutral
- Agree
- Strongly Agree

33.Responses of Gen AI tools do not reflect recent trends.

- Strongly Disagree
- Disagree
- Neutral
- Agree
- Strongly Agree

34.Responses of Gen AI tools don’t ignite fresh perspectives for an issue.

- Strongly Disagree
- Disagree
- Neutral
- Agree
- Strongly Agree

35.Responses by Gen AI tools don’t contain human qualities like empathy or human consciousness.

- Strongly Disagree
- Disagree
- Neutral
- Agree
- Strongly Agree

36.Responses by Gen AI tools contain human qualities like humor.

- Strongly Disagree
- Disagree
- Neutral
- Agree
- Strongly Agree

37.Responses of Gen AI tools violate intellectual property rights.

- Strongly Disagree
- Disagree
- Neutral
- Agree
- Strongly Agree

38.Gen AI Tools lead to plagiarism.

- Strongly Disagree
- Disagree
- Neutral
- Agree
- Strongly Agree

39.It is not clear who is legally responsible for content created by Gen AI.

- Strongly Disagree
- Disagree
- Neutral
- Agree
- Strongly Agree

40.Gen AI tools do not have legal responsibility.

- Strongly Disagree
- Disagree
- Neutral
- Agree
- Strongly Agree

41.Using Gen AI tools leads to defamation incidents.

- Strongly Disagree
- Disagree
- Neutral
- Agree
- Strongly Agree

42.The content of Gen AI tools belittles people.

- Strongly Disagree
- Disagree
- Neutral
- Agree
- Strongly Agree

43.What is your age in years.

- Below 30 years
- 30 to 45 yrs
- 46 to 60 yrs
- Above 60 yrs

44.What is your Gender?

- Male
- Female
- Non-binary
- Prefer not to say

45.What is your educational qualification?

- Under-graduate
- Graduate
- Post-graduate
- Others

46.What is your employment status?

- Salaried
- Self-employed
- Retired
- In Sabbatical Break to pursue education
- Left Job to pursue degree education

47.State your Nationality. Single line text.

----------------------------------------------------------------------------------------------------------------

48.State the country in which you pursued your highest level of education. Single line text.

----------------------------------------------------------------------------------------------------------------
